# A Non-Hazardous Deparaffinization Protocol Enables Quantitative Proteomics of Core Needle Biopsy-Sized Formalin-Fixed and Paraffin-Embedded (FFPE) Tissue Specimens

**DOI:** 10.3390/ijms23084443

**Published:** 2022-04-18

**Authors:** Georgia Mitsa, Qianyu Guo, Christophe Goncalves, Samuel E. J. Preston, Vincent Lacasse, Adriana Aguilar-Mahecha, Naciba Benlimame, Mark Basik, Alan Spatz, Gerald Batist, Wilson H. Miller, Sonia V. del Rincon, René P. Zahedi, Christoph H. Borchers

**Affiliations:** 1 Division of Experimental Medicine, McGill University, Montreal, QC H4A 3J1, Canada; georgia.mitsa@outlook.com (G.M.); qianyu.guo@rocketmail.com (Q.G.); mark.basik@mcgill.ca (M.B.); gerald.batist@mcgill.ca (G.B.); wilsonmiller@gmail.com (W.H.M.J.); sonia.delrincon@gmail.com (S.V.d.R.); 2 Segal Cancer Proteomics Centre, Lady Davis Institute, Jewish General Hospital, McGill University, Montreal, QC H3T 1E2, Canada; vincent.lacasse2@mail.mcgill.ca; 3 Gerald Bronfman Department of Oncology, Jewish General Hospital, McGill University, Montreal, QC H4A 3T2, Canada; goncalves.christophe@gmail.com (C.G.); samuel.preston@mail.mcgill.ca (S.E.J.P.); nanaaguilar@gmail.com (A.A.-M.); alan.spatz@mcgill.ca (A.S.); 4 Research Pathology Core Facility, Department of Pathology, Segal Cancer Centre, Lady Davis Institute, Jewish General Hospital, McGill University, Montreal, QC H3T 1E2, Canada; nbenlimame@jgh.mcgill.ca; 5 Exactis Innovation, 5450 Cote-des-Neiges, Suite 522, Montreal, QC H3T 1Y6, Canada; 6 Rossy Cancer Network, McGill University, Montreal, QC H3H 1E8, Canada

**Keywords:** clinical proteomics, tumor tissues, FFPE, quantitative proteomics, core needle biopsy, cancer research, molecular pathology, breast ductal carcinoma, in situ cancer

## Abstract

Most human tumor tissues that are obtained for pathology and diagnostic purposes are formalin-fixed and paraffin-embedded (FFPE). To perform quantitative proteomics of FFPE samples, paraffin has to be removed and formalin-induced crosslinks have to be reversed prior to proteolytic digestion. A central component of almost all deparaffinization protocols is xylene, a toxic and highly flammable solvent that has been reported to negatively affect protein extraction and quantitative proteome analysis. Here, we present a ‘green’ xylene-free protocol for accelerated sample preparation of FFPE tissues based on paraffin-removal with hot water. Combined with tissue homogenization using disposable micropestles and a modified protein aggregation capture (PAC) digestion protocol, our workflow enables streamlined and reproducible quantitative proteomic profiling of FFPE tissue. Label-free quantitation of FFPE cores from human ductal breast carcinoma in situ (DCIS) xenografts with a volume of only 0.79 mm^3^ showed a high correlation between replicates (r^2^ = 0.992) with a median %CV of 16.9%. Importantly, this small volume is already compatible with tissue micro array (TMA) cores and core needle biopsies, while our results and its ease-of-use indicate that further downsizing is feasible. Finally, our FFPE workflow does not require costly equipment and can be established in every standard clinical laboratory.

## 1. Introduction

Most human tumor tissues that are obtained for pathology and diagnostic purposes are formalin-fixed and paraffin-embedded (FFPE) [1]. FFPE allows the preservation of tissues in a “life-like” state while preserving spatial features and keeping them accessible for subsequent downstream analyses, such as immunohistochemistry (IHC), and genomic (hotspot) sequencing, without the requirement of expensive equipment for sample storage [2,3,4,5]. Vast FFPE tissue archives are available in clinics around the globe. These represent an invaluable resource for precision oncology and clinical research [1,6] because the archives often include pathological, clinical, and outcome data that are linked to the clinical samples, which have often been collected from a patient during different stages of disease.

The MS-based proteomic analysis of FFPE samples has gained increasing attention during the past decade, not only because of improved protocols for the extraction of proteins, but also because of substantial improvements in the overall sensitivity of MS instrumentation and workflows [7]. These technological advances now enable the proteomic profiling of minute sample amounts. Both system-wide ‘discovery’ data on aberrant protein expression/signaling pathway activity and targeted data providing actual protein concentrations for selected protein targets can provide important phenotypic information from FFPE samples that cannot be extracted from genomic screening or from IHC staining, and which may be in disagreement with genomic information [8,9,10].

Nevertheless, quantitative proteomics of FFPE samples, i.e., minuscule FFPE cores (down to 1 mm in diameter) or thin FFPE slices (down to 5 µm thick), which are key to utilizing the full potential of FFPE proteomics, are still far from routine. This can be partially attributed to the challenges in obtaining a *well-defined* and *homogenous* tissue sample, which may require micro-/macro-dissection to enrich for the area of interest following examination by a pathologist of a hematoxylin and eosin (H&E) stained representative slide. Once a well-defined FFPE sample has been obtained, paraffin has to be removed in order to make the sample amenable to MS-based proteomics, as paraffin interferes with the subsequent steps of proteomic sample preparation and MS analysis [11]. In addition, formalin-induced crosslinks have to be reversed prior to protein extraction, which is then typically followed by a bottom-up proteomic workflow. A central component of almost all deparaffinization protocols is xylene, which is a toxic and highly flammable solvent [12,13]. It has also been reported that xylene may negatively affect protein extraction and quantitative proteomic analysis [3,14]. Most studies agree that heat, the choice of detergents and protein denaturants, as well as the pH of the extraction buffer and physical agitation, are important parameters affecting the efficacy of protein extraction [3,15]. 

**Figure 1:**
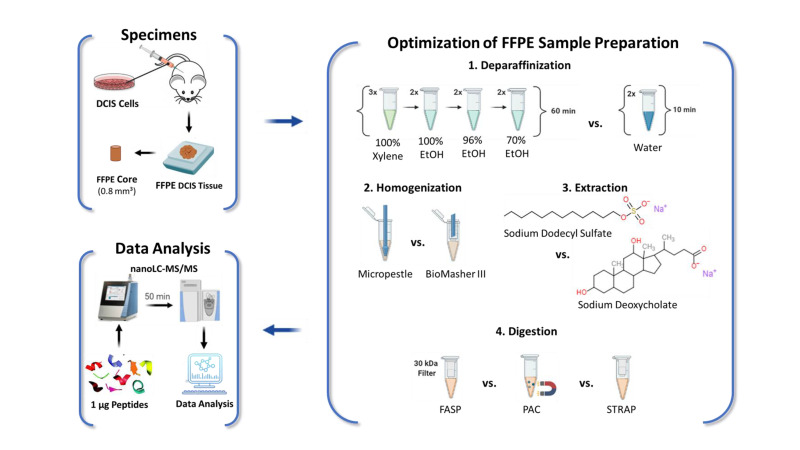
Experimental Design. Xenografts were generated from human DCIS cells and tumors were resected after 1.5 weeks, followed by formalin-fixation and paraffin-embedding, as described in [17]. One-millimeter-diameter FFPE cores were used to optimize individual steps of the FFPE sample preparation: (1) deparaffinization, (2) homogenization, (3) extraction, and (4) digestion. Peptide samples were analyzed by nano-LC-MS/MS label-free quantitation (LFQ) to compare the performance of the evaluated protocols for each step of the sample preparation workflow.

To make quantitative proteomics of FFPE cores and slides more streamlined and easier to automate for use in the clinic, as well as to expand its use beyond the current focus on retrospective studies, we present here an accelerated and efficient workflow for FFPE proteomics that does not require xylene and which can be set up in any standard clinical laboratory, as it requires neither costly equipment nor special training (Figure 1). The absence of xylene in our protocol is in line with the principles of “green chemistry” [16] as it avoids the use of chemicals that are both hazardous to nature and involve a hazardous synthesis.

## 2. Results

### 2.1. Water-Based Deparaffinization Competes with the Gold-Standard Xylene and Takes Only a Fraction of the Time

The initial step, common to all FFPE sample preparation protocols, is deparaffinization, and the protocol used in most laboratories is essentially the reversal of the paraffinization procedure, comprising many steps that cannot be readily automated and are time-consuming: e.g., sequential washing steps with xylene and decreasing concentrations of ethanol (100%, 96%, and 70%). Our goal was to develop a simpler and safer protocol. We, therefore, compared the standard xylene-procedure to a deparaffinization method that is based on washes with hot water (*depW*) without toxic solvents. The use of water for deparaffinization had been first suggested in 2016 by Kalantari et al. for DNA extraction [18], and 2017 by Mansour et al. for Western blot analysis [14].

After deparaffinization with either xylene (*depX*) or hot water (*depW*), the samples were homogenized using a disposable micropestle (*homMP*) in a sodium deoxycholate (SDC)-based buffer (*exSDC)* and digested using *FASP*. Details can be found in Section 4.3.1.

**Figure 2:**
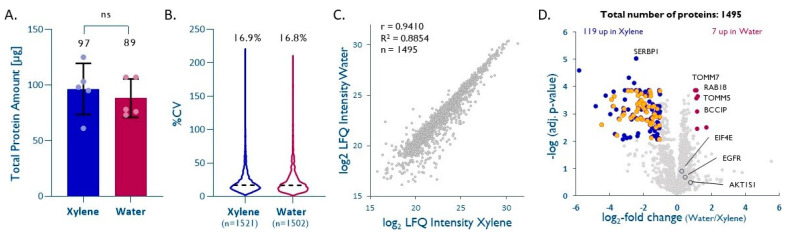
Water-based deparaffinization is a ‘green’ alternative. (**A**) Total protein extracted after deparaffinization with either water (*depW*) or xylene (*depX*) (*n* = 5, unpaired *t*-test, *p* = 0.54). (**B**) Intra-method %CVs based on quantified proteins, median %CV are given. (**C**) Pearson correlation plot based on all proteins quantified by both methods. (**D**) Volcano plot highlighting proteins significantly enriched by either method (Benjamini–Krieger multiple hypothesis testing, FDR 1%). Cytosolic ribosomal proteins significantly enriched with *depX* are shown in orange. EIF4E, EGFR, and AKT1S1 are highlighted in dark grey.

Our data show that two cycles of short incubation (~5 min) in hot water is sufficient to efficiently retrieve proteins from FFPE cores, thus avoiding laborious successive washes with organic solvents (8 steps, 60 min). Water-based deparaffinization (*depW*) yielded, on average, 89 ± 17 µg of total protein per mg (dry weight) of FFPE core, compared to 97 ± 23 µg using xylene (*depX*; unpaired *t*-test *p* = 0.54, Figure 2A).

Database searches performed on individual samples led to the identification of 933 ± 26 (*depX*) and 835 ± 80 (*depW*) proteins, 6778 ± 294 (*depX*) and 5925 ± 608 (*depW*) peptides, and 8400 ± 352 (*depX*) and 7244 ± 815 (*depW*) peptide-spectrum matches (PSM), respectively, from 23,008 ± 561 (*depX*) and 22,764 ± 1378 (*depW*), acquired MS/MS spectra. A quantitative comparison of the five *depW* and *depX* replicates using label-free quantitation (LFQ) led to the quantitation of 1502 (*depW*) and 1521 (*depX*) unique proteins across the five replicates, with intra-method CVs of 16.8% (*depW*) and 16.9% (*depX*; Figure 2B). The LFQ-derived intensities of 1495 proteins that were quantified between the two methods show a good agreement between *depW* and *depX* (correlation coefficient R = 0.94, slope = 0.97, Figure 2C). In general, the choice of the deparaffinization method did not significantly impact the recovery of hydrophobic or hydrophilic proteins (see Supplementary Table S1); however, almost half of the quantified high-abundance cytosolic ribosomal proteins showed poorer recoveries with *depW* (Figure 2D; Benjamini–Krieger adjusted *p* value <0.01, median *depW/depX* = 0.25), while membrane and nuclear proteins of interest in cancer biology, such as TOMM5 [19,20], TOMM7 [19,21], RAB18 (RAS related protein) [22,23], and nuclear BCCIP (BRCA2 interacting protein) [22,24], had significantly better recoveries using *depW* (adj. *p* < 0.01, *depW/depX*= >2.08). Notably, other important cancer proteins, such as EGFR (adj. *p* = 0.20, *depW/depX* = 1.36), EIF4E (adj. *p* = 0.13, *depW/depX* = 1.19), or AKT1S1 (adj. *p* = 0.31, *depW/depX* = 1.71) seem to show better recoveries using water-based deparaffinization, but this was not statistically significant.

### 2.2. Efficient Tissue Homogenization Using Micropestles

Next, we evaluated different tools for the homogenization of small tissue samples. We compared the total protein amounts extracted with a disposable, autoclavable micropestle (*homMP*) or a BioMasher III (*homBM*) which is a micropestle with a filter unit that is commonly used in genomics studies [25,26,27,28]. The samples were deparaffinized using *depW*, homogenized with either *homMP* or *homBM* in *exSDC* and digested using *FASP*. Details can be found in Section 4.3.1.

**Figure 3:**
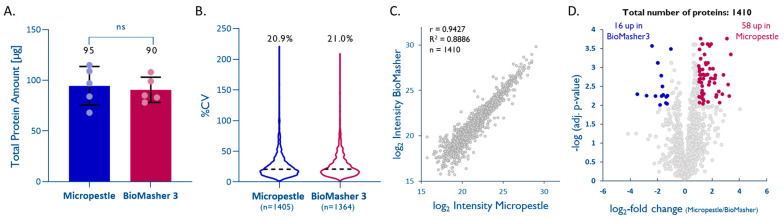
Efficient tissue homogenization using micropestles. (**A**) Total protein extracted from 1 mm cores with a dry-weight < 1 mg (*n* = 5; unpaired *t*-test; *p* = 0.70). (**B**) Intra-method %CVs based on all quantified proteins, median %CV are given. (**C**) Pearson correlation plot based on all quantified proteins. (**D**) Volcano plot highlighting proteins that were significantly enriched by one method (multiple hypothesis testing using the FDR-based approach by Benjamini–Krieger, FDR 1%).

Based on the bicinchoninic acid assay (BCA), both methods yielded similar protein amounts (95 ± 19 µg for *homMP* vs. 90 ± 13 µg for *homBM*; Figure 3A). LFQ of the five replicates per method enabling the quantitation of 1405 (*homMP*) and 1364 (*homBM*) unique proteins showed that both methods are equally reproducible (%CVs of 20.9% and 21.0% for *homMP and homBM*, respectively; Figure 3B). LFQ-derived normalized abundances of 1410 proteins that were quantified by both methods showed a good correlation (r = 0.94, slope = 0.98, Figure 3C), with a tendency toward higher intensities for *homMP* (Figure 3D). The proteins with significantly differential recovery (Figure 3D), however, do not seem to indicate a role of pI, hydrophobicity, subcellular localization, or molecular weight in the enrichment/depletion with either method (see Supplemental Table S2). Based on a slightly better overall performance, ease of use, and lower cost, we prefer the disposable micropestle to the BioMasher III.

### 2.3. Improved Protein Extraction with Sodium Deoxycholate (SDC)

After mechanical cell disruption using a disposable micropestle (*homMP*), preceded by deparaffinization using hot water (*depW*), the homogenate was incubated in different extraction buffers, and digested using *FASP*.

**Figure 4:**
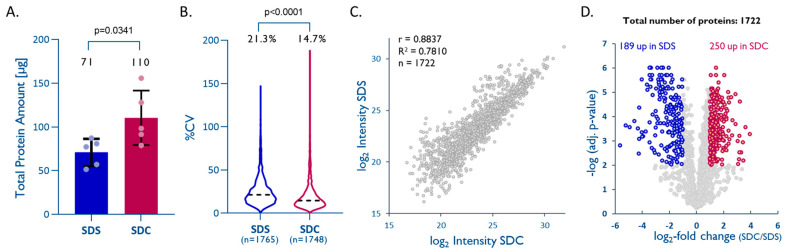
An SDC–TCEP-based buffer improves overall protein recovery from FFPE tissues. (**A**) Total protein extracted from 1 mm cores with dry-weight < 1 mg (*n* = 5; unpaired *t*-test; *p* = 0.0341). (**B**) Intra-method %CVs based on all quantified proteins, median %CVs are given (unpaired *t*-test; *p* < 0.0001). (**C**) Pearson correlation plot based on all proteins quantified with both methods. (**D**) Volcano plot highlighting proteins that were significantly enriched by either method (multiple hypothesis testing using FDR-based approach by Benjamini–Krieger, FDR 1%).

Formalin fixation of tissue preserves proteins in their native structures, but studies suggest that several modifications may occur during the fixation process, and these seem to progress over time [29,30,31]. Lysine methylation and methionine oxidation are the most frequent protein modifications observed in FFPE tissue [30,31]. These protein modifications can ultimately lead to protein–protein, DNA–protein, and/or RNA–protein crosslinking (e.g., Schiff base reaction). Combining heat [32,33] with high concentrations of detergents is considered to be the most effective approach for protein extraction from FFPE tissue and the reversal of protein-crosslinking that had been induced during the fixation process. We, therefore, compared a standard SDS buffer (*exSDS*), as used in many studies [3,15], to an SDC-based buffer (*exSDC*) [34,35,36]. *exSDC* contains TCEP, a potent denaturing agent, which seems to improve the denaturation of FFPE-preserved proteins and facilitates downstream processing. We use the identification of peptides with lysine methylation as an indicator of effective denaturation and decrosslinking in FFPE samples. We observed 4.95 ± 0.62% of these modified peptides with *exSDS* and 3.62 ± 0.26% with *exSDC*. This indicates that both lysis buffers are effective for decrosslinking, but *exSDC* seems to be marginally better. In our hands, the *exSDC* buffer yielded significantly higher amounts of total protein (unpaired *t*-test, *p* = 0.0341, Figure 4A) than the SDS buffer, which is thought to be the ‘gold-standard’.

LFQ data on 1748 (*exSDC*) and 1765 (*exSDS*) quantified unique proteins also showed a higher reproducibility for *exSDC* than for *exSDS* (%CV of 14.7% vs. 21.3%) (Figure 3B). A direct comparison using LFQ enabled the quantitation of 1722 unique proteins quantified by both methods and showed a slight tendency towards higher intensities, and consequently, are more likely to be a better extraction efficiency for *exSDC* (Figure 3C,D). A closer look into the physicochemical properties of proteins exclusively quantified in each method reveals that *exSDC* is significantly better for extraction of larger (unpaired *t*-test, *p* = 0.0001), more acidic (unpaired *t*-test, *p* = 0.005), and more hydrophilic proteins (unpaired *t*-test, *p* = 0.0001) than *exSDS* (see Supplemental Table S3).

### 2.4. PAC and STRAP Are Good Alternatives to FASP

Next, we evaluated the efficacy of different digestion techniques for FFPE-proteomics, namely, filter-aided sample preparation (*FASP*) [37,38], which is still one of the most-widely used sample preparation methods, in addition to the more recent protein-aggregation capture (*PAC*) [39] and suspension trapping using micro spin columns (*STRAP*) [40] that enable a simpler sample preparation with improved parallelization and automation capabilities [31,41,42,43,44]. For each method, 20 µg of total protein were digested after water-based deparaffinization, micropestle homogenization, and SDC-based extraction (*depW/homMP/exSDC).*

Important differences in the three digestion procedures are the (i) time and temperature of incubation with trypsin, and (ii) the substrate-to-protein ratio. While *FASP* typically involves overnight digestion at 37 °C, *PAC* and *STRAP* digestions are usually performed for 3 h at 37 °C and 47 °C, respectively. *FASP* and *PAC* samples were digested with a substrate-to-trypsin ratio of 20:1, while *STRAP* samples were digested with a substrate-to-trypsin ratio of 10:1, following the manufacturer’s instructions. Importantly, although it worked well for conventional samples, in our hands, the standard PAC protocol led to a poor performance for FFPE samples, which seemed to result from an overall poor recovery of proteins/peptides. We were able to compensate for this by adjusting the standard protein-to-bead ratio from 1:4 to 1:12, which led to substantially higher signals in the analysis, comparable to the other two methods. Notably, for STRAP, the buffer was brought to a final concentration of 5% SDS before loading, as recommended by the manufacturer.

**Figure 5:**
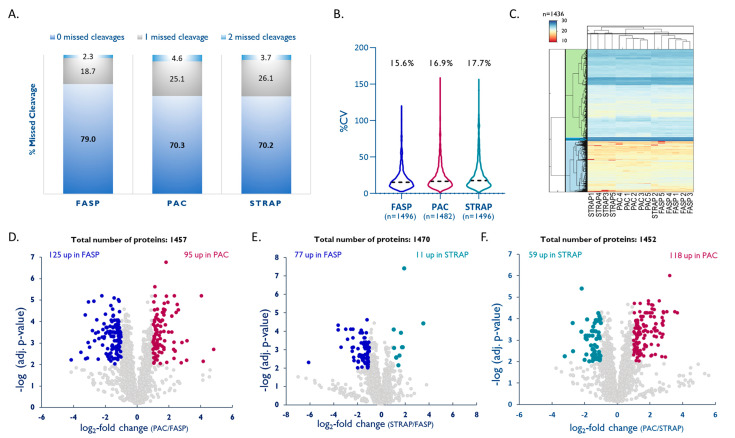
Comparison of PAC and STRAP with FASP. (**A**) Efficacy of tryptic digestion shown by percentage of missed cleavages. (**B**) Intra-method %CVs based on all quantified proteins, median %CVs are given. (**C**) Hierarchical clustering [49] of all quantified proteins, colors reflect log2-normalized abundances. (**D**–**F**) Volcano plots highlighting proteins that were significantly enriched by either method (multiple hypothesis testing using FDR-based approach by Benjamini–Krieger, FDR 1%).

When comparing the three digestion protocols, *FASP* yielded a clearly higher proportion of fully tryptic peptides (79%) compared to *PAC* and *STRAP* (both 70%; Figure 5A). LFQ of the individual methods showed that *FASP* also had the lowest intra-method %CV (15.6%, *n =* 1496) followed by *PAC* with 16.9% (*n* = 1482) and *STRAP* with 17.7% (*n* = 1496; Figure 5B). A quantitative comparison of the three methods based on LFQ led to the quantitation of 1436 unique proteins quantified by all methods. Unsupervised hierarchical clustering based on the normalized abundances of these proteins (multiple hypothesis testing using FDR-based approach by Benjamini–Krieger, FDR 1%, Figure 5C) shows good agreement between the three methods. A closer look into the physicochemical properties of proteins that were exclusively recovered with one of the methods shows that *PAC* seems to be better suited for small and more-acidic proteins, although not statistically significant (see Supplementary Table S4). A pair-wise comparison of the three methods by LFQ shows that *FASP* results in a slightly better overall protein recovery than *PAC*, while both methods are superior to *STRAP* (see Figure 5D–F).

Thus, PAC is a good alternative to the considerably more laborious and time-consuming *FASP*, is also easily scalable to the amount of protein, and has already been fully automated using liquid-handling systems [41,45,46,47,48].

## 3. Discussion

In this paper, we present a streamlined and efficient protocol for quantitative proteomics of FFPE cores based on a novel, ‘green’, and non-hazardous water-based method for deparaffinization (*depW*) prior to quantitative proteomic analysis, and show that harsh, non-MS compatible detergents, such as SDS, are not required for efficient retrieval of proteins from archived FFPE material.

The water-based deparaffinization results in paraffin-removal that is six times as fast as the conventional xylene-based protocol and enables robust proteomic profiling from less than 1 mg of FFPE core tissue (dry weight with wax) for clinical research. To our knowledge, this is the first report of a protocol for efficient protein retrieval from 1 mm-diameter core punches (~0.8 mm^3^ tissue volume). This volume is comparable to tissue micro array (TMA) cores used to build TMAs for IHC analysis, and also to core needle biopsies, thus enabling proteomic analysis of clinical samples that were not amenable to clinical research due to very limited tissue availability, i.e., ductal carcinoma in situ, where tumor areas of interest are too small for analyses beyond IHC.

Interestingly, several membrane receptors with central roles in cancer biology showed significantly better recoveries (*p* < 0.01, ≥2-fold) using our water-based method compared to xylene-based protocols (e.g., TOMM5/7, RAB18, BCCIP) [50]. Other relevant cancer targets, such as translational elongation and initiation factors (e.g., EIF4E), membrane receptors (e.g., EGFR), as well as RNA binding proteins, also showed a tendency towards better recovery, albeit not statistically significant. These results may reflect the differential impact of water- and several rounds of ethanol-washes on the partial removal of either soluble or hydrophobic proteins.

More recent protocols for proteomics of FFPE samples include the use of Adaptive Focused Acoustics (AFA) for efficient deparaffinization and decrosslinking [40,41,43,51], but the availability of AFA systems that can handle multiple samples in standard laboratories is limited because of the high cost of both the instrumentation and the required consumables. In our hands, sonication of the small sample amounts used in this study results in a high risk of sample loss, even when a single tube is used. A heat-based homogenization/lysis in SDC buffer with TCEP, together with autoclavable micropestles, enabled effective decrosslinking, homogenization, and extraction, using a simple protocol. A BCA kit compatible with reducing agents (RAC-BCA) is recommended to account for interferences in the colorimetric detection and quantitation of total protein.

The translation of FFPE-based proteomics into high-throughput (clinical) applications requires fast, reproducible, scalable, and (semi)automated workflows. We, therefore, performed a direct comparison of more recent techniques for protein extraction and digestion (i.e., PAC and STRAP), to the ‘gold-standard’ filter-assisted sample preparation (FASP).

Our data reveal differences in the efficiency of tryptic digestion and the feasibility for use on low sample amounts (scalability). Although FASP performs slightly better in our hands, it is a very laborious procedure including several washing steps that cannot be automated. It has been shown to work best for a protein range between 10 µg to 100 µg [52,53]. STRAP is a very attractive sample preparation method because the available cartridge formats cover a wide range of total protein amounts (1–100 µg, 100–300 µg or ≥300 µg) [40]. It also allows tryptic digestion in as little as 3 h, with comparable digestion efficiency to overnight digestion used for FASP. In our hands, however, STRAP was slightly less reproducible than PAC and FASP, with PAC being the best choice for very low sample amounts. PAC requires tryptic digestion for only 3 h and has already been successfully automated in several laboratories [41,45,46,47,48]. Moreover, a variety of bead-chemistries are available (e.g., amine-reactive, carboxylic, HILIC, etc.) which might be adapted based on specific research needs. Notably, to enable efficient PAC-based sample preparation for FFPE samples, we had to increase the recommended protein-to-bead ratio by a factor of three, likely as a result of FFPE-matrix effects that reduced the protein binding capacity.

## 4. Materials and Methods

We used FFPE tissue from human ductal breast carcinoma in situ (DCIS) xenografts to optimize the main steps of FFPE sample preparation: (i) deparaffinization, (ii) homogenization, (iii) protein extraction, and (iv) proteolytic digestion (see Figure 1). To avoid a systematic bias derived from tissue heterogeneity, we used 1 mm-diameter cores that had been obtained from a single FFPE block for each step (i–iv) of the protocol to be optimized, and randomly assigned these to the different protocols in order to have a total of 5 replicates per condition. Thus, any statistically significant differences observed should result from methodical differences rather than tissue heterogeneity.

Each method was evaluated based on the total protein yield (RAC-BCA), the number of identified peptides and proteins (qualitative MS), as well as the reproducibility and the enrichment/depletion of proteins (quantitative MS).

### 4.1. Chemicals and Reagents

All chemicals and reagents were purchased from Sigma Aldrich (St. Louis, Michigan, USA) unless otherwise stated. For sample homogenization, two types of micropestles were acquired, one from Sigma Aldrich (#BAF199230001) and one Optima Inc., Glencoe, IL, USA, (#320302). Filter-aided sample preparation (FASP) [37,38] was conducted on Microcon^®^ Centrifugal Filters (30 kDa molecular cut-off, Merck KGaA #MRCF0R030, purchased through Sigma Aldrich. For bead-based sample preparation, ferromagnetic beads with MagReSyn^®^ Amine functional groups (ReSyn Biosciences, Gauteng, South Africa) were used. For sample preparation using suspension trapping, S-Trap [51] micro-cartridges with a binding capacity of <100 µg total protein were purchased from ProtiFi (Farmingdale, NY, USA, #CO2-micro-80).

The total protein concentration was determined using a reducing-agent-compatible Pierce BCA Protein Assay Kit (Thermo Fisher Scientific, Waltham, MA, USA, #23250) following the manufacturer’s instructions.

### 4.2. Source of Specimens

**Figure 6:**
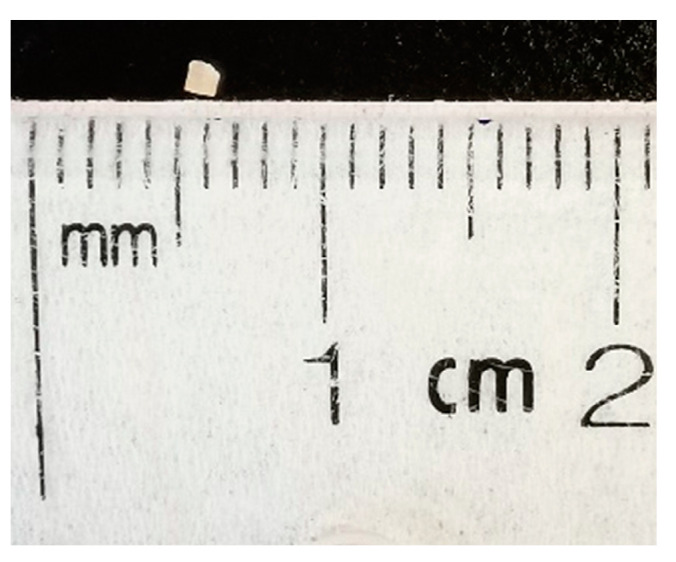
Representative size of FFPE core used in this study. After deparaffinization, the core volume was approximately 0.4 mm^3^.

Method development was performed using 1 mm-diameter FFPE cores (~0.8 mm^3^ tissue volume) of xenografts from human ductal breast carcinoma in situ (DCIS, Figure 6). A total of 1 × 10^5^ human DCIS cells (MCF10DCIS.com; Wake Forest University, Winston-Salem, NC, USA) were injected into the mammary fat pads of an athymic nude mouse. DCIS tumors were resected after 1.5 weeks, were formalin-fixed, paraffin-embedded, and stored under ambient conditions (~1 year) [17].

### 4.3. Sample Preparation of Core Needle Biopsy-Sized Specimens

#### 4.3.1. Optimization of Deparaffinization

Recovering proteins from FFPE tissue requires prior deparaffinization with the vast majority of protocols being based on xylene, followed by a series of washes with decreasing amounts of ethanol for tissue rehydration [2,31,42]. We compared a standard protocol for xylene/ethanol washes (*depX*) used in most clinical laboratories for paraffin removal, to a ‘green’ and solvent-free deparaffinization approach based on hot deionized water (80 °C; *depW*) using 5 FFPE cores per protocol.

**Figure 7:**
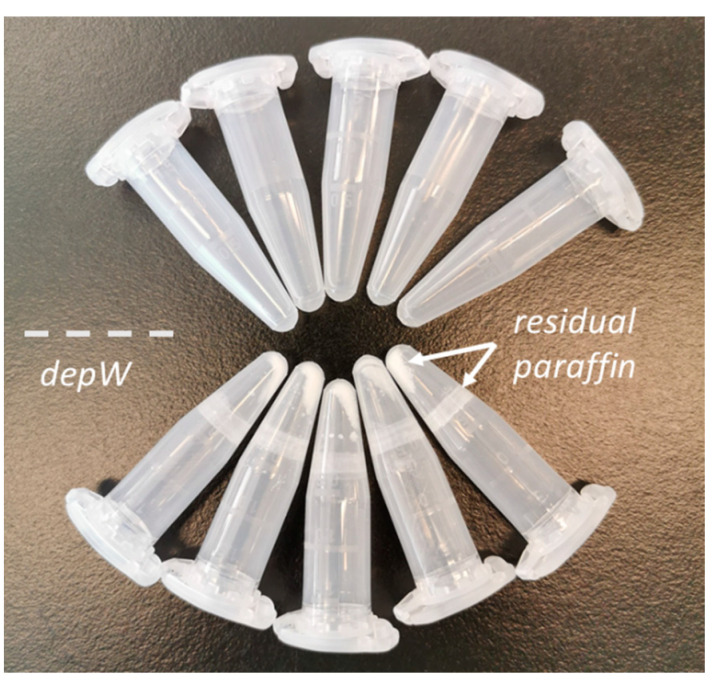
Representative tubes after deparaffinization. The molten paraffin in the *depW* approach forms a layer on the surface of the hot water and residual paraffin ‘flakes’ precipitate to the bottom of the tube during centrifugation. The deparaffinized tissue floats in the water and can be easily transferred into a new tube for further sample preparation.

(a) *depX* [54]: The samples were washed with 1 mL of 100% xylene and incubated for 10 min at room temperature (RT), followed by centrifugation at 14,000× *g* for 2 min and disposal of the supernatant, followed by another 2 repetitions. Then, the samples were washed twice each with 1 mL of 100%, 96%, and 70% ethanol, followed by incubation for 1 min at RT and centrifugation as above.
(b) *depW* (modified from [14]): The samples were washed 2× with 500 µL of hot deionized water and incubated for 1 min at RT under vigorous vortex mixing. Each washing step was followed by centrifugation at 20,000× *g* for 5 min at 4 °C. The supernatant, containing paraffin either floating on the liquid surface or stuck to the wall of the tube (Figure 7), was discarded and the deparaffinized and rehydrated core was transferred to a clean LoBind Eppendorf tube.

Each core was mechanically disrupted using a micropestle (Sigma Aldrich) in 250 µL of 2% sodium deoxycholate (SDC), 50 mM Tris-HCl, 10 mM tris(2-carboxyethyl)phosphine (TCEP), pH 8.5, followed by sequential incubation on an Eppendorf ThermoMixer C (purchased from VWR International, Mississauga, ON, CA) for 20 min at 99 °C (700 rpm) and for 2 h at 80 °C (900 rpm). The samples were cooled down on ice for 5 min, followed by RAC-BCA protein determination using 9 µL aliquots. Free cysteines were alkylated using 30 mM iodoacetamide (IAA) for 30 min at room temperature (RT), protected from light, followed by a quench with 10 mM dithiothreitol (DTT) for 15 min at RT. Tryptic digestion was performed by FASP [37,38] with slight modifications [55]. Briefly, lysate corresponding to 20 µg of total protein was diluted to 450 µL with freshly prepared 8 M Urea,100 mM Tris, pH 8.5 [56] and loaded onto a 30 kDa Microcon filter. The sample was centrifuged for 25 min at 13,500× *g* and the eluate was discarded, followed by three washes using 100 µL of the same buffer and three washes with 50 mM ammonium bicarbonate (AmBic). Finally, 100 µL of digestion buffer comprising 1:20 (*w*/*w*) trypsin:protein in 0.2 M guanidine-hydrochloride (GuHCl), 50 mM AmBic, 2 mM CaCl_2_ were added and the sample was incubated at 37 °C for 14 h. The tryptic peptides were recovered by centrifugation for 15 min at 13,500× *g*, followed by two additional washes using 50 µL of 50 mM AmBic and 50 µL of ultrapure water. The collected peptide sample was dried under vacuum and reconstituted in 0.1% formic acid (FA) for nano-LC-MS/MS.

#### 4.3.2. Optimization of Tissue Homogenization

250 µL of extraction buffer (2% SDC, 50 mM Tris-HCl, 10 mM TCEP, pH 8.5) were added to each H_2_O-deparaffinized (*depW*) tissue core (*n* = 10) for mechanical cell disruption and homogenization using either a disposable micropestle (*homMP*; Sigma Aldrich, #BAF199230001) or a BioMasher III (*homBM*; Optima Inc., #320302). After homogenization, the samples were digested using FASP as described above, dried under vacuum, and reconstituted in 0.1% FA for nano-LC-MS/MS.

#### 4.3.3. Optimization of Protein Extraction

Two buffers were compared: 4% SDS (*w*/*v*), 150 mM NaCl, 50 mM Tris-HCl, pH 8.5 (*exSDS*) [3,15,39] and 2% SDC, 50 mM Tris-HCl, 10 mM TCEP, pH 8.5 (*exSDC*). Then, 250 µL of either *exSDS* or *exSDC* buffer were added to each deparaffinized core. Samples were homogenized and digested as described for *homMP*. Samples were dried under vacuum and reconstituted in 0.1% FA for nano-LC-MS/MS.

#### 4.3.4. Optimization of Tryptic Digestion

Three strategies for proteolytic digestion were compared: FASP [1,2] using a 30 kDa MW cut-off filter [2,57], protein aggregation capture (PAC) [39], and suspension trapping using S-Trap micro-cartridges (STRAP) [40,51]. The cores were deparaffinized using H_2_O (*depW*) and homogenized using a disposable micropestle (*homMP*) in 2% SDC, 50 mM Tris-HCl, 10 mM TCEP, pH 8.5 (*exSDC*).

(a) *FASP* was performed as described above.
(b) *PAC* was performed using amine microparticles (MagReSyn) based on Batth et al. [39]. For 20 µg of protein lysate, 12 µL of microparticle stock solution (20 µg/mL) were equilibrated with 100 µL of 70% ACN, briefly vortexed and placed on a magnetic rack to remove the supernatant. This step was repeated another two times. Next, the protein extracts were added to the beads and the sample was adjusted to a final concentration of 70% ACN, thoroughly vortexed and incubated for 10 min at RT without shaking. The following washing steps were performed on a magnetic rack without disturbing the protein/bead aggregate. The supernatants were discarded, and the beads were washed on the magnetic rack with 1 mL of 95% ACN for 10 s, followed by a wash with 1 mL of 70% ACN without disturbing the protein/bead aggregate. The tubes were removed from the magnetic rack, 100 µL of digestion buffer (1:20 (*w*/*w*) trypsin:protein in 0.2 M GuHCl, 50 mM AmBic, 2 mM CaCl_2_) were added and the samples were incubated at 37 °C for 3 h. After acidification with trifluoroacetic acid (TFA) to a final concentration of 2%, the tubes were placed on the magnetic rack for 1 min, followed by removal of the supernatant. To remove residual beads, the samples were centrifuged at 20,000× *g* for 10 min. The supernatants were dried under vacuum and reconstituted in 0.1% FA for nano-LC-MS/MS.
(c) *STRAP* digestion was performed according to the manufacturer’s instructions [51]. Lysate corresponding to 20 µg of total protein was acidified to a final concentration of 1.2% phosphoric acid. SDS was added to a final concentration of 2% followed by a 7-fold dilution with STRAP binding buffer (90% methanol, 100 mM Tris-HCl, pH 7.1). The sample was loaded onto the STRAP and centrifuged at 4000× *g* for 1 min, followed by three washes with 150 µL binding buffer, with the spin-column being rotated by 180° between centrifugation steps. Then, 200 µL of STRAP digestion buffer, comprised of 1:10 (*w*/*w*) trypsin:protein in 0.2 M GuHCl, 50 mM AmBic, 2 mM CaCl_2_ were added to the STRAP, which was briefly spun on a benchtop centrifuge to assure saturation of the column material with the digestion buffer. The flow-through was loaded again on top of the column. The sample was incubated at 47 °C for 3 h. Peptides were eluted by sequential elution (1000xg, 1 min) using 40 µL of 50 mM AmBic, 40 µL of 0.1% FA, and 35 µL of 50% ACN, 0.1% FA. The collected peptide sample was dried under vacuum and reconstituted in 0.1% FA for nano-LC-MS/MS.

### 4.4. Data Analysis

All samples were analyzed by data dependent acquisition (DDA) using an Easy-nLC 1200 (Thermo Fisher Scientific, Waltham, MA, USA) coupled to a Q Exactive Plus (Thermo Fisher Scientific) mass spectrometer that was operated with a Nanospray Flex ion source (Thermo Fisher Scientific). To minimize systematic errors, all samples from one experimental set (e.g., comparison of *FASP/PAC/STRAP*) were injected in a randomized order. Then, 1 µg of digested protein were preconcentrated on an AcclaimPepMap 100 C18 pre-column (Thermo Fisher Scientific, 3 µm particle size, 75 µm inner diameter × 2 cm length) and separated on an AcclaimPepMap 100 C18 main column (Thermo Fisher Scientific, 2 µm particle size, 75 µm inner diameter x 25 cm length) using a 50 min binary gradient (A: 0.1% FA; B: 84% ACN in 0.1% FA) at a flow rate of 300 nL/min. B was increased from 3–17% until min 30 and from 17–40% until min 20. Full MS scans were acquired from *m*/*z* 350–1500 at a resolution of 70,000 with an automatic gain control (AGC) target value of 1 × 10^6^ and a maximum injection time of 50 ms. The 15 most intense precursor ions (charge states +2, +3, +4) were isolated with a window of *m*/*z* 1.2 and fragmented using a normalized collision energy of 28; the dynamic exclusion was set to 40 s. MS/MS spectra were acquired at a resolution of 17,500, using an AGC target value of 2 × 10^4^ and a maximum injection time of 64 ms.

MS raw data were processed using Proteome Discoverer 2.4 (PD, Thermo Fisher Scientific). Database searches were performed using SequestHT and a human Swissprot database (January 2019; 20,414 target entries). Label-free quantitation (LFQ) was performed using the Minora feature detector node, Percolator was used to calculate posterior error probabilities. Database searches were performed using trypsin as enzyme with a maximum of 2 missed cleavages. Carbamidomethylation of cysteine (+57.021 Da) was set as fixed modification and oxidation of methionine (+15.995 Da), and lysine methylation (+14.016 Da, +28.031 Da, and +42.047 Da) as variable modifications [31]. Mass tolerances were set to 5 ppm for precursor- and 0.02 Da for product-ions. The data were filtered to a false discovery rate (FDR) < 1% on the peptide and protein levels. Only proteins that were (i) identified with at least two unique peptides and (ii) quantified in at least 3 out of 5 replicates of at least one of the methods to be compared, were considered for the quantitative comparison. Protein LFQ data obtained from Proteome Discoverer were normalized based on summed protein intensities to correct for differences in sample loading. For proteins passing the abovementioned criteria, missing protein intensity values were imputed using 1.5× the minimum observed intensity for this particular sample. The obtained normalized abundances were used for unpaired *t*-tests (two tailed, 95% confidence) and Pearson correlation analyses. Differential expression analysis was performed on log2-transformed normalized abundance data with multiple hypothesis testing using a false discovery approach by Benjamini–Krieger false discovery rate (FDR 1%). Proteins having q-values of <0.01 and absolute log2 fold-changes >1 were considered as differential between tested workflows. Statistical analysis was performed using GraphPad Prism 9 (San Diego, CA, USA).

MS data files are publicly available through the ProteomeXchange Consortium via the PRIDE partner repository [58] with the dataset identifier PXD031946.

## 5. Conclusions

Based on our results, we recommend the following protocol for quantitative proteomics of small FFPE core samples: (i) water-based deparaffinization (*depW*) followed by homogenization using a micropestle (*homMP*) in an SDC-based extraction buffer containing reducing agents, e.g., DTT or TCEP (*exSDC*). In particular, for protein amounts of <10 µg, we recommend PAC-based digestion, while amounts of up to 100 µg work well with FASP. Importantly, based on our results, the tendency for better recoveries of subsets of proteins with certain properties might further influence the choice of sample preparation—for example, small and more acidic proteins showed better recoveries using PAC (see Supplemental Table S4).

This ’green’ FFPE proteomics workflow requires less starting material than used in comparable studies, i.e., <1 mg dry-weight of a FFPE core with wax, and enables robust proteomic profiling of FFPE tumor tissues (average RSD <20%) for clinical research. The micro-volumes of FFPE tissue used successfully in this study show that our workflow is well-suited for small tissue samples preserved in FFPE blocks such as core needle biopsies, and also small tissue cores such as those used to build TMAs. Diseases where tissue areas of interest are very limited, e.g., in pre-invasive cancer, are currently only amenable to histological assessment for diagnostic purposes. Our protocol enables clinical research and molecular characterization of such diseases, as only 1 mm in diameter cores are required. Furthermore, our workflow does not require any special equipment, is suitable for any standard hospital clinical laboratory, and can be automated, thereby facilitating high-throughput analysis for clinical research.

We have already used this protocol successfully for quantitative proteomics of FFPE cores and sections in breast cancer patient-derived xenografts for precise quantitation of PTEN [59], AKT1/2, and PIK3CA (publication in preparation), as well as in clinical samples of non-small cell lung cancer (NSCLC) for quantitative assessment of proteins in the PDL1-axis (publication in preparation). Tissue areas of interest as small as ~1 mm^3^ and, based on the total amounts of extracted protein, even considerably smaller volumes of FFPE tissues should be compatible with this workflow.

## Data Availability

MS data files are publicly available through the ProteomeXchange Consortium via the PRIDE partner repository [59] with the dataset identifier PXD031946.

## Supplementary Materials

The following supporting information can be downloaded at: https://www.mdpi.com/article/10.3390/ijms23084443/s1.

## References

[1] Gaffney E.F., Riegman P.H., Grizzle W.E., Watson P.H. (2018). Factors that drive the increasing use of FFPE tissue in basic and translational cancer research. Biotech. Histochem..

[2] Foll M.C., Fahrner M., Oria V.O., Kuhs M., Biniossek M.L., Werner M., Bronsert P., Schilling O. (2018). Reproducible proteomics sample preparation for single FFPE tissue slices using acid-labile surfactant and direct trypsinization. Clin. Proteom..

[3] Maes E., Broeckx V., Mertens I., Sagaert X., Prenen H., Landuyt B., Schoofs L. (2013). Analysis of the formalin-fixed paraffin-embedded tissue proteome: Pitfalls, challenges, and future prospectives. Amino Acids.

[4] Maes E., Valkenborg D., Mertens I., Broeckx V., Baggerman G., Sagaert X., Landuyt B., Prenen H., Schoofs L. (2013). Proteomic analysis of formalin-fixed paraffin-embedded colorectal cancer tissue using tandem mass tag protein labeling. Mol. Biosyst..

[5] Ralton L.D., Murray G.I. (2011). The use of formalin fixed wax embedded tissue for proteomic analysis. J. Clin. Pathol..

[6] Seyhan A.A., Carini C. (2019). Are innovation and new technologies in precision medicine paving a new era in patients centric care?. J. Transl. Med..

[7] Sobsey C.A., Ibrahim S., Richard V.R., Gaspar V., Mitsa G., Lacasse V., Zahedi R.P., Batist G., Borchers C.H. (2019). Targeted and Untargeted Proteomics Approaches in Biomarker Development. Proteomics.

[8] Alvarez-Chaver P., De Chiara L., Martinez-Zorzano V.S. (2018). Proteomic Profiling for Colorectal Cancer Biomarker Discovery. Methods Mol. Biol..

[9] Choi C.H., Chung J.Y., Kang J.H., Paik E.S., Lee Y.Y., Park W., Byeon S.J., Chung E.J., Kim B.G., Hewitt S.M. (2020). Chemoradiotherapy response prediction model by proteomic expressional profiling in patients with locally advanced cervical cancer. Gynecol. Oncol..

[10] Toomey S., Carr A., Mezynski M.J., Elamin Y., Rafee S., Cremona M., Morgan C., Madden S., Abdul-Jalil K.I., Gately K. (2020). Identification and clinical impact of potentially actionable somatic oncogenic mutations in solid tumor samples. J. Transl. Med..

[11] Faoláin E.Ó., Hunter M.B., Byrne J.M., Kelehan P., Lambkin H.A., Byrne H.J., Lyng F.M. (2005). Raman spectroscopic evaluation of efficacy of current paraffin wax section dewaxing agents. J. Histochem. Cytochem..

[12] Kandyala R., Raghavendra S.P.C., Rajasekharan S.T. (2010). Xylene: An overview of its health hazards and preventive measures. J. Oral Maxillofac. Pathol..

[13] European Chemical Agency Xylene (Last Updated: 16 April 2020). https://echa.europa.eu/brief-profile/-/briefprofile/100.014.124.

[14] Mansour A.G., Khalil P.A., Bejjani N., Chatila R., Dagher-Hamalian C., Faour W.H. (2017). An optimized xylene-free protein extraction method adapted to formalin-fixed paraffin embedded tissue sections for western blot analysis. Histol. Histopathol..

[15] Magdeldin S., Yamamoto T. (2012). Toward deciphering proteomes of formalin-fixed paraffin-embedded (FFPE) tissues. Proteomics.

[16] Anastas P.T., Warner J.C. (1998). Green Chemistry: Theory and Practice.

[17] Guo Q., Li V.Z., Nichol J.N., Huang F., Yang W., Preston S.E.J., Talat Z., Lefrere H., Yu H., Zhang G. (2019). MNK1/NODAL Signaling Promotes Invasive Progression of Breast Ductal Carcinoma In Situ. Cancer Res..

[18] Kalantari N., Bayani M., Ghaffari T. (2016). Deparaffinization of formalin-fixed paraffin-embedded tissue blocks using hot water instead of xylene. Anal. Biochem..

[19] The Human Protein Atlas. https://www.proteinatlas.org/.

[20] Maertens A., Tran V.P., Maertens M., Kleensang A., Luechtefeld T.H., Hartung T., Paller C.J. (2020). Functionally Enigmatic Genes in Cancer: Using TCGA Data to Map the Limitations of Annotations. Sci. Rep..

[21] Sekine S., Wang C., Sideris D.P., Bunker E., Zhang Z., Youle R.J. (2019). Reciprocal roles of Tom7 and OMA1 during mitochondrial import and activation of PINK1. Mol. Cell.

[22] Alnouti Y. (2009). Bile Acid Sulfation: A Pathway of Bile Acid Elimination and Detoxification. Toxicol. Sci..

[23] Zhong K., Chen K., Han L., Li B. (2014). MicroRNA-30b/c inhibits non-small cell lung cancer cell proliferation by targeting Rab18. BMC Cancer.

[24] Meng X., Liu J., Shen Z. (2003). Genomic structure of the human BCCIP gene and its expression in cancer. Gene.

[25] Fujikawa T., Miyata S.-I., Iwanami T. (2013). Convenient detection of the citrus greening (huanglongbing) bacterium ‘Candidatus Liberibacter asiaticus’ by direct PCR from the midrib extract. PLoS ONE.

[26] Alqaydi M., Roy R. (2016). Quantitative and qualitative study of STR DNA from ethanol and formalin fixed tissues. Forensic Sci. Int..

[27] Yamamoto T., Nakashima K., Maruta Y., Kiriyama T., Sasaki M., Sugiyama S., Suzuki K., Fujisaki H., Sasaki J., Kaku-Ushiki Y. (2012). Improved RNA extraction method using the BioMasher and BioMasher power-plus. J. Vet. Med. Sci..

[28] Yamamoto T., Ushiki Y., Hara S., Hall W.W., Tsukagoshi-Nagai H., Yokoyama T., Tagawa Y., Sata T., Yamakawa Y., Kinoshita N. (2008). An advantageous method utilizing new homogenizing device BioMasher and a sensitive ELISA to detect bovine spongiform encephalopathy accurately in brain tissue. J. Virol. Methods.

[29] Zhang Y., Muller M., Xu B., Yoshida Y., Horlacher O., Nikitin F., Garessus S., Magdeldin S., Kinoshita N., Fujinaka H. (2015). Unrestricted modification search reveals lysine methylation as major modification induced by tissue formalin fixation and paraffin embedding. Proteomics.

[30] Sprung R.W., Brock J.W.C., Tanksley J.P., Li M., Washington M.K., Slebos R.J.C., Liebler D.C. (2009). Equivalence of Protein Inventories Obtained from Formalin-fixed Paraffin-embedded and Frozen Tissue in Multidimensional Liquid Chromatography-Tandem Mass Spectrometry Shotgun Proteomic Analysis. Mol. Cell. Proteom..

[31] Coscia F., Doll S., Bech J.M., Schweizer L., Mund A., Lengyel E., Lindebjerg J., Madsen G.I., Moreira J.M., Mann M. (2020). A streamlined mass spectrometry–based proteomics workflow for large-scale FFPE tissue analysis. J. Pathol..

[32] Metz B., Kersten G.F.A., Baart G.J.E., de Jong A., Meiring H., ten Hove J., van Steenbergen M.J., Hennink W.E., Crommelin D.J.A., Jiskoot W. (2006). Identification of Formaldehyde-Induced Modifications in Proteins:  Reactions with Insulin. Bioconjugate Chem..

[33] Shi S.R., Liu C., Balgley B.M., Lee C., Taylor C.R. (2006). Protein extraction from formalin-fixed, paraffin-embedded tissue sections: Quality evaluation by mass spectrometry. J. Histochem. Cytochem..

[34] Proc J.L., Kuzyk M.A., Hardie D.B., Yang J., Smith D.S., Jackson A.M., Parker C.E., Borchers C.H. (2010). A quantitative study of the effects of chaotropic agents, surfactants, and solvents on the digestion efficiency of human plasma proteins by trypsin. J. Proteome Res..

[35] Lin Y., Lin H., Liu Z., Wang K., Yan Y. (2014). Improvement of a sample preparation method assisted by sodium deoxycholate for mass-spectrometry-based shotgun membrane proteomics. J. Sep. Sci..

[36] Erde J., Loo R.R., Loo J.A. (2014). Enhanced FASP (eFASP) to increase proteome coverage and sample recovery for quantitative proteomic experiments. J. Proteome Res..

[37] Wisniewski J.R., Zougman A., Nagaraj N., Mann M. (2009). Universal sample preparation method for proteome analysis. Nat. Methods.

[38] Manza L.L., Stamer S.L., Ham A.J., Codreanu S.G., Liebler D.C. (2005). Sample preparation and digestion for proteomic analyses using spin filters. Proteomics.

[39] Batth T.S., Tollenaere M.A.X., Rüther P.L., Gonzalez-Franquesa A., Prabhakar B.S., Bekker-Jensen S.H., Deshmukh A.S., Olsen J.V. (2019). Protein aggregation capture on microparticles enables multi-purpose proteomics sample preparation. Mol. Cell. Proteom..

[40] Wilson J., Mayyappan V., Narducci D.N., Neely B., Laugharn J., Pappin D. (2018). Universal Sample Processing of Multiple Sample Types for Reproducible Proteomic Sample Preparation. HUPO.

[41] Müller T., Kalxdorf M., Longuespée R., Kazdal D.N., Stenzinger A., Krijgsveld J. (2020). Automated sample preparation with SP3 for low-input clinical proteomics. Mol. Syst. Biol..

[42] Buczak K., Ori A., Kirkpatrick J.M., Holzer K., Dauch D., Roessler S., Endris V., Lasitschka F., Parca L., Schmidt A. (2018). Spatial tissue proteomics quantifies inter- and intra-tumor heterogeneity in hepatocellular carcinoma. Mol. Cell. Proteom..

[43] Schweizer L., Coscia F., Müller J., Doll S., Wierer M., Mann M. AFA-sonication Followed by Modified Protein Aggregation Capture (APAC) Enables Direct, Reproducible and Non-toxic Sample Preparation of FFPE Tissue for Mass Spectrometrybased Proteomics. Covaris Appl. Note-M020141.

[44] Hughes C.S., Moggridge S., Müller T., Sorensen P.H., Morin G.B., Krijgsveld J. (2019). Single-pot, solid-phase-enhanced sample preparation for proteomics experiments. Nat. Protoc..

[45] Stoychev S., Govender I., Naicker P., Gerber I., Jordaan J., Pauw M., Tabb D., Arribas Diez I., Norregaard Jensen O., Baath T. (2019). Development of a fully automated high throughput magnetic workflow for phosphoproteome profiling. HUPO.

[46] Tape C.J., Worboys J.D., Sinclair J., Gourlay R., Vogt J., McMahon K.M., Trost M., Lauffenburger D.A., Lamont D.J., Jørgensen C. (2014). Reproducible automated phosphopeptide enrichment using magnetic TiO2 and Ti-IMAC. Anal. Chem..

[47] Martínez-Val A., Bekker-Jensen D.B., Steigerwald S., Stoychev S., Gerber I., Jordaan J., Bache N., Olsen J.V. Fast and reproducible phosphoproteomics using MagReSyn Amine and Ti-IMAC HP magnetic beads and the Evosep One. Tech. Note.

[48] Leutert M., Rodriguez-Mias R.A., Fukuda N.K., Villén J. (2019). R2-P2 rapid-robotic phosphoproteomics enables multidimensional cell signaling studies. Mol. Syst. Biol..

[49] Nolte H., MacVicar T.D., Tellkamp F., Krüger M. (2018). Instant Clue: A Software Suite for Interactive Data Visualization and Analysis. Sci. Rep..

[50] The Human Proteome Atlas. https://www.proteinatlas.org.

[51] Marchione D.M., Ilieva I., Devins K., Sharpe D., Pappin D.J., Garcia B.A., Wilson J.P., Wojcik J.B. (2020). HYPERsol: High-Quality Data from Archival FFPE Tissue for Clinical Proteomics. J. Proteome Res..

[52] Sielaff M., Kuharev J., Bohn T., Hahlbrock J., Bopp T., Tenzer S., Distler U. (2017). Evaluation of FASP, SP3, and iST Protocols for Proteomic Sample Preparation in the Low Microgram Range. J. Proteome Res..

[53] Wiśniewski J.R. (2019). Filter Aided Sample Preparation—A tutorial. Anal. Chim. Acta.

[54] Boellner S., Becker K.F. (2015). Reverse Phase Protein Arrays-Quantitative Assessment of Multiple Biomarkers in Biopsies for Clinical Use. Microarrays.

[55] Shema G., Nguyen M.T.N., Solari F.A., Loroch S., Venne A.S., Kollipara L., Sickmann A., Verhelst S.H.L., Zahedi R.P. (2018). Simple, scalable, and ultrasensitive tip-based identification of protease substrates. Mol. Cell. Proteom..

[56] Kollipara L., Zahedi R.P. (2013). Protein carbamylation: In vivo modification or in vitro artefact?. Proteomics.

[57] Tanca A., Abbondio M., Pisanu S., Pagnozzi D., Uzzau S., Addis M.F. (2014). Critical comparison of sample preparation strategies for shotgun proteomic analysis of formalin-fixed, paraffin-embedded samples: Insights from liver tissue. Clin. Proteom..

[58] Vizcaino J.A., Deutsch E.W., Wang R., Csordas A., Reisinger F., Rios D., Dianes J.A., Sun Z., Farrah T., Bandeira N. (2014). ProteomeXchange provides globally coordinated proteomics data submission and dissemination. Nat. Biotechnol..

[59] Ibrahim S., Lan C., Chabot C., Mitsa G., Buchanan M., Aguilar-Mahecha A., Elchebly M., Poetz O., Spatz A., Basik M. (2021). Precise quantitation of PTEN by immuno-MRM: A tool to resolve the breast cancer biomarker controversy. Anal. Chem..

